# A Study on the Structural and Digestive Properties of Rice Starch–Hydrocolloid Complexes Treated with Heat–Moisture Treatment

**DOI:** 10.3390/foods12234241

**Published:** 2023-11-24

**Authors:** Yu Zhang, Boxin Dou, Jianhui Jia, Ying Liu, Na Zhang

**Affiliations:** 1 College of Food Engineering, Harbin University of Commerce, Harbin 150028, China; zhylcc8113855@163.com (Y.Z.); dbx0803@163.com (B.D.); jianhuijia1025@163.com (J.J.); zhangna@hrbcu.edu.cn (N.Z.); 2 College of Life Science and Technology, Mudanjiang Normal University, Mudanjiang 157011, China

**Keywords:** rice starch, xanthan gum, locust bean gum, HMT

## Abstract

Rice starch-hydrophilic colloid complexes (SHCs) were prepared by incorporating xanthan gum and locust bean gum into natural rice starch. Subsequently, they underwent hygrothermal treatment (H-SHC) to investigate their structural and digestive properties with varying colloid types and added amounts of H-SHC. The results demonstrated that heat–moisture treatment (HMT) led to an increase in resistant starch (RS) content in rice starch. This effect was more pronounced after the addition of hydrophilic colloid, causing RS content to surge from 8.42 ± 0.39% to 38.36 ± 3.69%. Notably, the addition of locust bean gum had a more significant impact on enhancing RS content, and the RS content increased with the addition of hydrophilic colloids. Enzyme digestion curves indicated that H-SHC displayed a lower equilibrium concentration (C_∞_), hydrolysis index (HI), and gluconeogenesis index (eGI). Simultaneously, HMT reduced the solubility and swelling power of starch. However, the addition of hydrophilic colloid led to an increase in the solubility and swelling power of the samples. Scanning electron microscopy revealed that hydrophilic colloid encapsulated the starch granules, affording them protection. X-ray diffraction (XRD) showed that HMT resulted in the decreased crystallinity of the starch granules, a trend mitigated by the addition of hydrophilic colloid. Infrared (IR) results demonstrated no formation of new covalent bonds but indicated increased short-range ordering in H-SHC. Rapid viscosity analysis and differential scanning calorimetry indicated that HMT substantially decreased peak viscosity and starch breakdown, while it significantly delayed the onset, peak, and conclusion temperatures. This effect was further amplified by the addition of colloids. Rheological results indicated that H-SHC displayed lower values for G′, G″, and static rheological parameters compared to natural starch. In summary, this study offers valuable insights into the development of healthy, low-GI functional foods.

## 1. Introduction

Rice serves as a staple food for over half of the global population, supplying essential energy and nutrients. Rice starch comprises approximately 90% of the dry weight of rice and includes both straight-chain and highly branched chain starch. Notably, the content of straight-chain starch in rice starch typically ranges from 0 to 40% [1]. While rice starch offers advantages such as fine granules, non-allergenic properties, and strong acid resistance, it does have limitations. These include easy digestion, rapid absorption, and a high glycemic index (GI), which poses a significant risk to individuals with chronic conditions like diabetes and obesity [2].

Englyst et al. [3] classify starch into rapidly digestible starch, slowly digestible starch (SDS), and resistant starch (RS) based on its in vitro digestion rate. The rate of starch digestion is closely linked to the risk of developing metabolic disorders like type 2 diabetes, obesity, and cardiovascular disease [4]. Rapidly digestible starch [5] leads to a sharp increase in the GI, elevating the risk of metabolic disorders. Conversely, SDS exhibits a milder impact on GI values, while RS resists digestion, serving as a fermentable substrate for intestinal flora and offering hypoglycemic and anti-obesity benefits. The content of RDS, SDS, and RS in starch is a pivotal factor affecting the GI, with RDS content positively correlated and SDS and RS content negatively correlated with the GI. Research indicates that postprandial glucose levels should be maintained within the range of 4–5.5 mmol/L to support normal organ and tissue metabolism. Elevated postprandial glucose levels, even within the normal range, can raise plasma insulin and lipid levels, potentially causing vascular endothelial dysfunction and increasing the risk of diabetes and cardiovascular diseases [6]. To enhance the digestive properties of rice starch, researchers commonly employ physical [7], chemical [7], and enzyme [8] modifications. HMT is a prevalent physical modification method used by researchers because it only involves water and heat, making it environmentally friendly [9]. HMT entails treating starch for a specific duration at lower moisture levels (10% to 30%) and higher temperatures (90 to 130 °C) [10]. HMT reduces starch’s swelling capacity and solubility [11,12], raises the pasting temperature, lowers peak viscosity, and degrades [1,13] and modifies the crystalline structure, particle morphology, and thermal properties of starch [14,15]. Moreover, HMT can alter the conformation of straight-chain and branched-chain starches, their interactions, and their arrangement within starch granules [16]. Hydrophilic colloids typically refer to macromolecules that readily dissolve in water and hydrate sufficiently to form viscous or gel-like solutions [17]. Mixing hydrophilic colloids with starch affects pasting properties, rheology, texture, viscoelasticity, and starch [18,19,20] digestibility. Additionally, mixing starch with hydrophilic colloids can modify starch digestibility by encapsulating starch particles or increasing the viscosity of the mixture [21]. The type [22] and quantity of colloids added [23] also influence starch GI and RS content [24]. Xanthan gum and locust bean gum possess distinct structures [25,26] implying potential variations in their effects on starch properties.

At this stage, the research mainly focuses on adding hydrophilic colloids only or heat–moisture treatment alone to improve the digestive resistance of starch. In this study, the two were combined to make starch and hydrophilic colloid bind more closely, and the digestive resistance of starch was effectively improved with the addition of a small amount of hydrophilic colloid. The study examined the impact of various types of colloids and their respective quantities on the structural characteristics of heat–moisture-treated hydrophilic colloid–rice starch complexes. Properties such as digestive resistance, crystal structure, short-range ordering, pasting performance, dynamic rheology, and steady-state rheology were thoroughly investigated, and the mechanism of their effects on the digestive resistance of starch was explored. The results of this study are expected to fill the knowledge gap on the impact of heat–moisture treatment combined with locust bean gum and xanthan gum on the digestive properties, pasting properties, and crystal structure of rice starch, promote the understanding of the inter-action between hydrophilic colloids and rice starch, and develop other new applications.

## 2. Materials and Methods

### 2.1. Materials

Rice: Paddy rice, sourced from Heilongjiang Province Wuchang Golden Harvest Rice Industry Limited Liability Company, Wuchang, China. Xanthan gum and locust bean gum: Biochemical reagents, obtained from Beijing Solebo Technology Co. Ltd., Beijing, China. Porcine pancreatic α-amylase e (12 U/mg) and amyloglucosidase (*Aspergillus niger*, 82.5 U/mg): Sourced from Sigma, St Louis, MO, USA. Glucose assay kits: GOPOD Format Acquired from Megazyme Inc., Bray, Ireland.

### 2.2. Extraction of Rice Starch

Rice starch was extracted with a slight modification to the method by Chen et al. [27]. The process involved crushing rice powder through a 100-mesh sieve, followed by the addition of a 0.3% NaOH solution. The solid–liquid ratio of 1:4 was maintained, and stirring occurred for 4 h. Subsequently, centrifugation at 3000× *g* for 15 min was performed, with the subsequent discarding of the supernatant. The precipitate was then washed with deionized water until the pH of the supernatant reached ≤7.0. The precipitate underwent mixing with anhydrous ethanol in a 1:3 ratio, stirring for 2 h, followed by centrifugation and removal of the supernatant. This washing, centrifugation, drying, and pulverization process was repeated three times. The centrifuged precipitate was then dried at 45 °C, pulverized, passed through a 100-mesh sieve, and sealed for storage. The amylose content of rice starch was 16.21%.

### 2.3. HMT Treatment of Samples

To perform the HMT of the samples, hydrophilic colloids (0 g, 0.1 g, 0.3 g, 0.5 g) were dispersed in 100 mL of deionized water and stirred in a water bath at 90 °C for 30 min. After cooling to room temperature, 10 g of starch (dry basis) was added and stirred for an additional 30 min. The samples were subsequently dried at 45 °C, pulverized to pass through a 100-mesh sieve, and their moisture content was adjusted to 25%. The samples were allowed to equilibrate for 24 h at 4 °C. They were then subjected to treatment in a reactor (KH-200 Kuncheng Co., Shanghai, China) at 120 °C for 5 h. After cooling to room temperature, the samples were stored at 4 °C for an additional 24 h. Finally, the samples were dried in an oven at 45 °C for 24 h, crushed through a 100-mesh sieve, and sealed in containers for storage.

### 2.4. Determination of RS Content

RS content was determined following the method described by Englyst et al. [28]. Briefly, a 100 mg starch sample was suspended in 25 mL of the mixed enzyme solution (3600 U pancreatic α-amylase and 360 U amyloglucosidase), followed by shaking at 37 °C in a water bath. An aliquot from the reaction mixture was taken out at 20 and 120 min, respectively, and a glucose assay kit was used to determine its glucose content. The RDS, SDS, and RS fractions in the starch sample were measured using the following formulas:

(1) $$RDS\%=\frac{\left(G_{20}-FG\right)\times 0.9}{TS}\times 100\%$$

(2) $$SDS\%=\frac{\left(G_{120}-G_{20}\right)\times 0.9}{TS}\times 100\%$$

(3) $$RS\%=\frac{TS-\left(RDS+SDS\right)}{TS}\times 100\%$$

where FG is the free glucose content (mg); G_20_ and G_120_ are the amounts of glucose released in 20 and 120 min of hydrolysis, respectively (mg); 0.9 represents the glucose conversion coefficient; and TS represents the total starch content in the sample (mg).

### 2.5. GI Measurement

The measurement of the GI involved determining the starch hydrolysis rate. This was achieved using a modified method inspired by Liu et al. [29]. The reaction solution was sampled at intervals of 0, 20, 40, 60, 90, 120, and 180 min, and its glucose content was analyzed using a glucose assay kit (GOPOD Format). Hydrolysis curves were constructed with the starch hydrolysis rate on the vertical axis and time on the horizontal axis.

These results were fitted to a first-order kinetics equation (Equation (4)) to calculate C_∞_ and k:

*C_t_* = *C*_∞_ × (1 − *e*^−*kt*^) (4)

where Ct is the concentration at t time; C_∞_ is the equilibrium concentration (t 180); and k is the hydrolysis equilibrium constant:

(5) $$AUC=C_{\infty }\left(t_{f}-t_{0}\right)-\frac{C_{\infty }}{k}\left[1-e^{-k\left(t_{f}-t_{0}\right)}\right]$$

where the AUC (area under curve) is the area under the hydrolysis curve, t_f_ is the final time (180 min), and t_0_ is the initial time.

The starch hydrolysis index (HI) of the samples was calculated from the area under the starch hydrolysis curve (AUC _samples_ and AUC _white bread_) of each sample from 0 to 180 min, using white bread (AUC _white bread_ = 134.99) as a reference. The estimated gluconeogenic index (eGI) of the samples was determined according to Equations (6) and (7).

(6) $$\mathrm{HI}=\frac{AUCsamples}{AUCwhite\;bread}\times 100$$

eGI = 8.198 + 0.862 × HI(7)

### 2.6. Observation of Particle Morphology

Particle morphology was observed using a scanning electron microscope (FEI Ltd., Hillsboro, OR, USA). Conductive double-sided adhesive was applied to the aluminum carrier stage of the microscope. A small amount of dried sample particles was adhered to the double-sided adhesive, coated with gold, and observed at a voltage of 15 kV and a magnification of 3000 times, with images captured using the scanning electron microscope.

### 2.7. X-ray Diffraction (XRD)

Samples were milled and subsequently passed through a 200-mesh sieve. An X-ray diffractometer (Rint-2000, Rigaku Co., Tokyo, Japan) was employed for measurement. The following test parameters were applied: a measurement voltage of 40 kV, a current of 40 mA, a diffraction angle (2θ) ranging from 5° to 40°, a scanning speed of 5°/min, and a step size of 0.02°.

### 2.8. Fourier Transform Infrared (FTIR)

To assess the short-range ordered structure of the samples, a Fourier Transform Infrared Spectrometer (Spectrum 100, Perkin Elmer Co., Waltham, MA, USA) was utilized. Dried samples were mixed with potassium bromide at a mass ratio of 1:50, followed by milling. The resulting mixture was pressed and subjected to Fourier transform infrared (FTIR) spectroscopy. Scanning was conducted within the range of 400–4000 cm^−1^, with a resolution of 4 cm^−1^.

### 2.9. Measurement of Pasting Characteristics

Pasting characteristics were determined using a Rapid Viscosity Analyzer (Rapid-20, Perten Co., Stockholm, Sweden). Starch solutions with a mass fraction of 14% (on a dry basis) were prepared through moisture correction according to the software accompanying the RVA. The samples were initially preheated at 50 °C for 1 min, then heated to 95 °C at a rate of 12 °C/min, held for 2 min, and subsequently cooled to 50 °C at the same rate and held for 2 min. Analysis was conducted using the TCW (3.17) software, and key parameters, including peak viscosity, trough viscosity, final viscosity, breakdown, and setback, were obtained.

### 2.10. Thermal Characterization

The thermal properties of starch samples were analyzed using a differential scanning calorimeter (DSC 4000, Perkin Elmer Co., Waltham, MA, USA). Deionized water was added at a sample-to-water ratio (m:m) of 1:2, sealed, and left to equilibrate at room temperature for 24 h. An empty crucible was employed as a control for the DSC determination. The temperature range spanned 20 to 150 °C, with a heating rate of 10 °C/min, and a nitrogen flow rate of 50 mL/min.

### 2.11. Determination of Solubility and Swelling Power

Solubility and swelling power were determined following the method described by Xie et al. [30]. A specific quantity (M) of the sample was accurately weighed and mixed with an appropriate amount of distilled water to prepare a starch solution with a mass fraction of 2%. The magnetic stirrer was set to temperatures sequentially at 60 °C, 70 °C, 80 °C, and 90 °C. Subsequently, the starch solution was heated and stirred for 30 min. Afterward, the sample was poured into a centrifuge tube and centrifuged at 4500× *g* for 10 min. The supernatant was collected, dried at 105 °C until reaching constant weight, and its mass (m) was weighed. The weight of the precipitate was noted as m1. Solubility (%) and swelling power (g/g) were then calculated using the following equations.

Solubility (%) = m/M × 100(8)

Swelling power (g/g) = m_1_/(M − m) (9)

### 2.12. Rheological Characterization

#### 2.12.1. Dynamic Rheology

The starch paste prepared in Section 2.9 was placed on a test sample stage of a rheometer (H-PID200, Waters Co., New Castle, DE, USA) using a 50 mm diameter plate with a set gap of 1 mm. Dynamic oscillatory rheological properties were determined at a constant temperature of 25 °C. The analysis covered a constant strain of 1% over a frequency range from 0.1 to 20.0 Hz, and dynamic rheological data, including energy storage modulus (G′), loss modulus (G″), and loss tangent (tan δ), were recorded.

#### 2.12.2. Static Rheology

After completing the determination of dynamic rheological properties, a 50 mm diameter plate was used with the gap set to 1 mm. Static rheological properties were assessed at a constant temperature of 25 °C. The shear rate ranged from 0.1 s^−1^ to 300 s^−1^, and the relationship between shear stress (σ) and shear rate (γ) was determined. The data were fitted with the Herschel–Bulkley equation shown in Equation (10):

τ = τ_0_ + K × γ*^n^* (10)

where τ is the shear stress, Pa; τ_0_ is the yield stress, Pa; K is the consistency coefficient, Pa-Sn; γ is the shear rate, s^–1^; and *n* is the fluid index.

### 2.13. Statistics and Analysis of Data

Data obtained from three independent measurements were expressed as means ± standard deviation. The data were analyzed with one-way analysis of variance (ANOVA), followed by Duncan’s multiple range test using SPSS 25.0 Statistical Software Program (SPSS Incorporated, Chicago, IL, USA). A significance level of (*p* < 0.05) was considered statistically significant. Graphs illustrating the data were generated using Origin 2021.

## 3. Results and Discussion

### 3.1. In Vitro Digestibility Analysis

Table 1 displays the RDS, SDS, and RS content of natural starch and HMT-treated samples. In comparison to natural rice starch, HMT-treated starch exhibited increased RDS content, decreased SDS content, and significantly higher RS content. These findings align with studies conducted by Chung on corn, pea, and lentil starch, highlighting the substantial impact of branched amylose structures, interactions, and internal starch molecule reconfiguration formed during HMT on RDS, SDS, and RS starch contents [31].

The RS content of H-SHC exhibited a positive correlation with the amount of colloid addition. However, the difference between 3% and 5% colloid addition was not significant. This may be attributed to a saturation point where a certain amount of colloid interacted fully with the starch, and excess colloid did not provide additional protection for H-SHC. Notably, the RS content of H-SLB was slightly higher than that of H-SXG, primarily due to locust bean gum’s superior hydrophilicity compared to xanthan gum. This observation aligns with the SEM results, which revealed differences in colloid adhesion to the starch. It further supports the notion that colloids in H-SHC encapsulate starch granules, hindering the contact between digestive enzymes and starch, thus protecting the starch granules, inhibiting enzymatic action, and ultimately increasing RS content. Additionally, hydrophilic colloids limited enzyme access to starch by increasing sample viscosity and promoting high water utilization, thereby reducing the rate of starch hydrolysis [32]. Moreover, the high viscosity of the gel reduces the activity of hydrolysis products, resulting in localized high product concentrations that inhibit digestive enzyme activity [33]. Slaughter et al. [34] demonstrated that hydrophilic colloids exhibit some affinity for a region of α-amylase, leading to non-competitive inhibition with starch.

H-NS represents samples of natural starch after wet heat treatment; H-SLB1% represents samples of rice starch mixed with 1% locust bean gum and subjected to wet heat treatment; H-SLB3% represents samples of rice starch mixed with 3% locust bean gum and subjected to wet heat treatment; H-SLB5% represents samples of rice starch mixed with 5% locust bean gum and subjected to wet heat treatment; H-SXG1% represents rice starch mixed with 1% xanthan gum and subjected to moist heat treatment; H-SXG3% indicates the sample of rice starch mixed with 3% xanthan gum and subjected to moist heat treatment; H-SXG5% indicates the sample of rice starch mixed with 5% xanthan gum and subjected to moist heat treatment.

**Figure 1:**
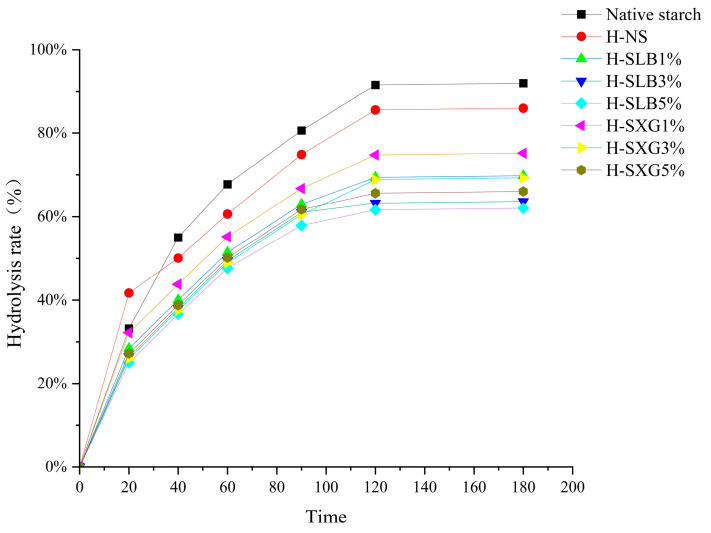
Hydrolysis curves of natural starch and HMT samples.

The hydrolysis curves of the samples are shown in Figure 1. The results of the first-level kinetic model fit to the hydrolysis curve are summarized in Table 1. HMT led to significant reductions in the equilibrium concentration of starch (C_∞_), hydrolysis index (HI), and gluconeogenesis index (eGI). These findings indicate that HMT substantially reduces starch digestibility. The addition of hydrophilic colloid further reduced these parameters. Notably, locust bean gum exhibited a significantly stronger inhibitory effect on enzymatic hydrolysis compared to xanthan gum. This inhibitory effect increased as the colloid amount increased from 1% to 5%. These results are consistent with in vitro starch digestion outcomes.

### 3.2. Scanning Electron Microscopy Analysis

**Figure 2:**
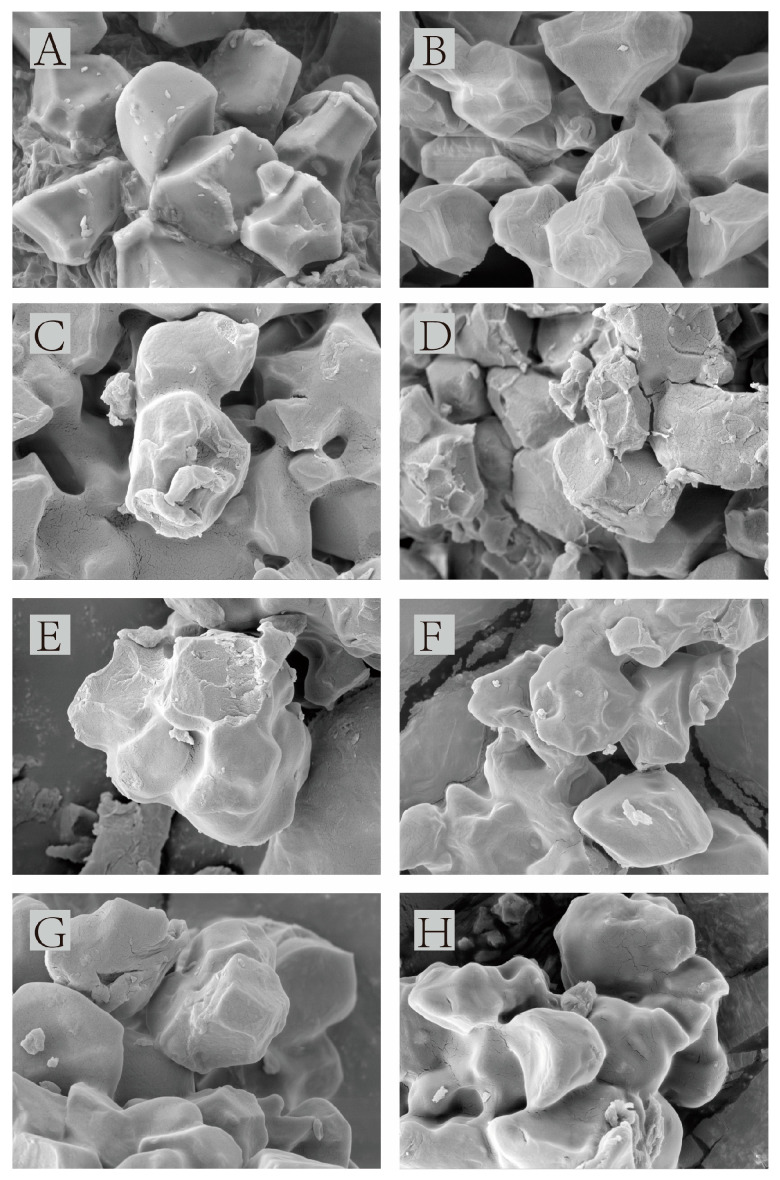
Scanning electron micrographs of natural starch and HMT samples: (**A**) natural starch, (**B**) H-NS, (**C**) H-SLB1%, (**D**) H-SLB3%, (**E**) H-SLB5%, (**F**) H-SXG1%, (**G**) H-SXG3%, (**H**) H-SXG5%.

The morphology of the samples was observed via SEM (Figure 2). Natural starch, after heat treatment, did not display significant changes in granule morphology or granule integrity. However, the presence of cracks and indentations indicated that thermal forces during HMT affected granule organization, highlighting the impact of pressure and heating on starch granules [35]. The addition of hydrophilic colloids resulted in noticeable differences in the morphology of starch granules compared to the original starch. As hydrophilic colloids were added, their attachment to the surface of starch granules increased gradually. The colloids exhibited a stretched, composite conformation, enhancing their pseudoplasticity. During mixing with starch, colloids underwent “shear thinning” and swelling, leading to uniform dispersion in the emulsion and adherence to starch granule surfaces. At a 1% colloid addition, the morphology of starch granules did not differ significantly from that of HMT starch, with evident cracks and indentations on the surface. However, when the colloid addition reached 3% and 5%, it was observed that starch granule surfaces exhibited prominent colloid attachment, accompanied by the disappearance of cracks and indentations. This indicated that colloids effectively protected starch granules during the HMT process.

Locust bean gum, composed of irregular galactose-branched mannan backbone chains, has a tendency to form intermolecular hydrogen bonds and curls. Although locust bean gum can interact with straight-chain starch molecules, it reduces the number of hydroxyl groups capable of forming amphiphilic hydrogen bonds with these starch molecules [36]. Consequently, xanthan gum adheres more uniformly compared to locust bean gum.

### 3.3. X-ray Diffraction Analysis

**Figure 3:**
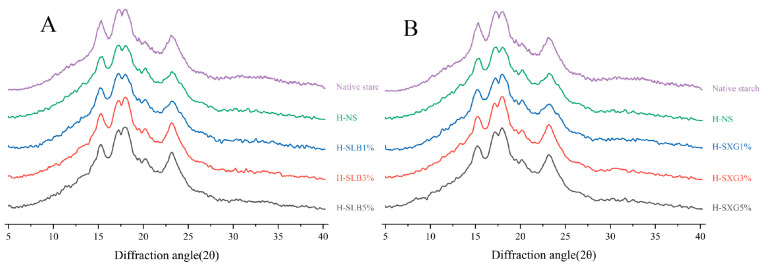
XRD patterns of natural starch and HMT samples: (**A**) XRD spectrum of H-SLB; (**B**) XRD spectrum of H-SXG.

The X-ray diffractograms of natural starch and HMT samples are presented in Figure 3. Natural starch exhibited strong reflections at 15.31, 17.31, 17.99, and 23.21. The strong reflection region of HMT, with the addition of hydrophilic colloid, closely resembled that of the original starch. Both natural starch and H-SHC displayed A-type crystalline structures. Table 2 reveals a slight decrease in the relative crystallinity of natural starch after HMT. This suggests a reduction in the crystallization of the semi-crystalline layer or an increase in the amorphous region. The primary reason for this decrease in crystallinity induced by HMT is likely the disruptive effect of the high temperature and high water content on the crystal structure. Under such conditions, the double helix structure of the crystalline region may undergo partial damage and rearrangement into a new structure, resulting in reduced relative crystallinity [9]. At 1% colloid addition, there was no significant difference in the crystallinity of H-SHC compared to natural starch under HMT. However, the relative crystallinity of H-SHC was notably higher than that of H-NS but not significantly different from that of natural starch at 3% and 5% colloid addition. This suggests that both locust bean gum and xanthan gum, under HMT, have a protective effect on the crystal structure of starch. Due to the hydrophilic nature of colloids, locust bean gum, and xanthan gum absorbed water from the crystalline region of starch during the melting process and encapsulated it on the surface of starch granules. This prevented water molecules from damaging the crystalline region of starch. The protective effect on the crystalline structure of starch granules was not significantly different between xanthan gum and locust bean gum.

### 3.4. Fourier Transform Infrared (FTIR) Spectral Analysis

**Figure 4:**
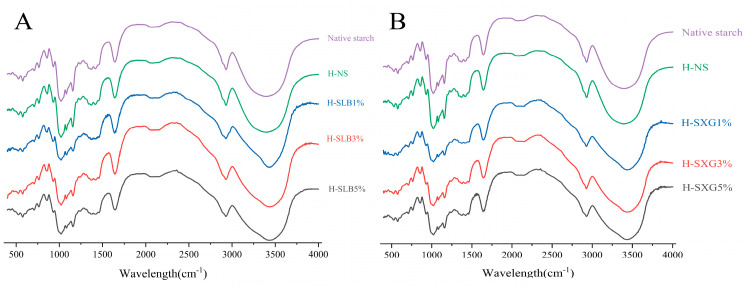
Infrared plots of natural starch and HMT samples: (**A**) IR absorption spectra of H-SLB; (**B**) IR absorption spectra of H-SXG.

Figure 4 displays the deconvolution spectra of the samples and natural starch within the 400–4000 cm^–1^ range. Comparing the spectra of natural starch and H-SHC reveals strong and broad absorption peaks in the range of 3300–3700 cm^–1^, likely attributed to the O-H stretching vibrations of the glucose unit [37]. Similar results have been found in yam flour and starch [38]. Although there was no significant change in the number of peaks in each sample after HMT compared to natural starch, there was a difference in the intensity of the peaks. This suggests that HMT does not affect the molecular composition, and no new covalent bonds form between starch and colloid. Instead, the bonding between the two polymers is of the hydrogen bonding type.

The 1047/1022 cm^–1^ absorbance ratios for all samples are provided in Table 2. This ratio indicates the short-range ordering of starch molecules [39], reflecting the state of the double helix organization related to the arrangement resulting from the interaction of straight-chain amylose, straight-chain amylose-branched amylose, and straight-chain amylose-branched amylose chains [40]. Slightly higher 1047/1022 ratios for H-NS compared to natural starch suggest a more efficient double helix ordering arrangement in the outer regions of the starch granules during HMT, indicating a higher degree of short-range molecular ordering. These ratios increased with the addition of colloid, significantly so at 3% and 5% colloid addition. This suggests that colloids effectively affect the double helix arrangement and can maintain the short-range ordering of rice starch. Xanthan gum displayed significantly lower 1047/1022 ratios than locust bean gum at the same concentration, indicating that locust bean gum is more capable of improving the short-range ordering of starch granules, consistent with the in vitro digestion results.

### 3.5. Analysis of Pasting Properties

**Figure 5:**
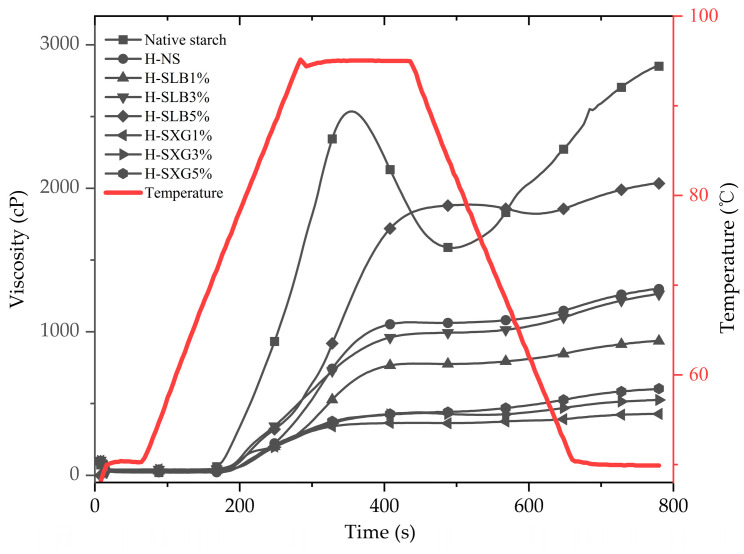
Pasting curves of natural starch and HMT samples.

Table 3 and Figure 5 presents the pasting properties of natural starch and HMT samples. The increase in starch pasting temperature may be attributed to HMT disrupting the original ordered structure of starch chains and rearranging them into a more tightly packed state, requiring more energy to break this enhanced structure during pasting [16]. HMT also reduced the peak viscosity and setback of starch, possibly due to the hydrolysis of some branched-chain starches into straight-chain starches, resulting in shorter chains with lower molecular mass and decreased peak viscosity, making the starch more difficult to regenerate [41]. Additionally, the addition of hydrocolloids reinforced these trends, with increased pasting temperatures and further reductions in peak viscosity and setback. Xanthan gum had a more pronounced effect than locust bean gum, indicating the improved shear stability of starch. This increased stability rendered the starch less prone to disintegration, making it more resistant to enzymatic hydrolysis and digestion [14].

### 3.6. Thermal Performance Analysis

The thermodynamic properties of natural starch and HMT samples are summarized in Table 4. DSC results revealed that HMT significantly delayed the onset pasting temperature (T_0_), peak pasting temperature (Tp), and conclusion temperature [42] of post-HMT starch, and the range of pasting temperatures (Tc − T_0_) was significantly increased. This suggests the formation of a more stable crystal structure via H-NS; in fact, it is known that the enhanced starch double helix structure requires higher temperatures to be disrupted [31]. The formation of this stronger structure may be due to the rearrangement of starch chains and the redistribution of water molecules during the HMT process [41]. It has been reported that this rearrangement converts a large fraction of SDS into RS [43]. The addition of hydrophilic colloids further delayed T_0_, Tp, and Tc values, with a wider delay observed with increasing colloid addition. This indicates that hydrophilic colloids inhibited starch granule pasting, which is consistent with previous reports [44]. The greater hydration capacity of hydrocolloids reduced the mobility of water molecules, similar to the effect of reduced water content [45], reducing water uptake in the amorphous starch zone and leading to increased T0, Tp, and Tc values.

The gelatinization enthalpy (ΔH) of H-NS was slightly lower compared to natural starch. This decrease may be attributed to the instability of the crystals, requiring less energy to break them. High temperatures increase the mobility of the double helices forming the crystal structure, leading to the breaking of hydrogen bonds between neighboring double helices. The ΔH of H-NS was lower than that of natural starch [46]. However, the addition of hydrophilic colloids significantly increased the ΔH of the samples, and the effect of colloid type on the change in ΔH was not significant. However, the amount of colloid added had a significant effect on the ΔH, which showed an increase with increasing amount of colloid. This is due to the fact that starch-exposed hydrogen bonding and colloid polymerization form two continuous gel network structures inhibiting starch pasting [47]. This indicates that colloids not only encapsulated the surface of starch granules but also interacted with straight-chain starch through hydrogen bonding in the granular state. This interaction contributed to the decreased digestibility of H-SHC.

### 3.7. Swelling Power and Solubility Analysis

**Figure 6:**
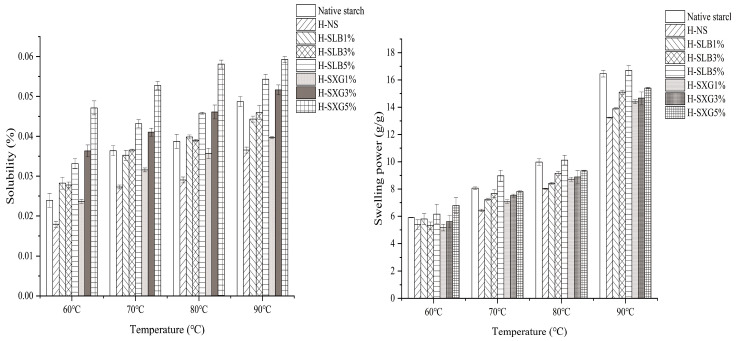
Solubility and dissolution of natural starch and HMT samples at different temperatures.

The swelling power and solubility of natural starch with H-SHC samples at temperatures of 60 °C, 70 °C, 80 °C, and 90 °C are depicted in Figure 6. The swelling power of all samples increased as the temperature rose. However, the swelling power of natural starch decreased following HMT treatment, which aligns with findings from previous studies [35]. This decline can be attributed to alterations in the morphology, composition, and interactions of the starch granule components. It is also associated with increased interactions between straight-chain and branched-chain starch molecules, leading to strengthened intramolecular bonds [14]. Furthermore, the decrease in swelling power can be attributed to enhanced interactions between straight-chain and branched-chain starch molecules, the formation of straight-chain starch–lipid complexes, and changes in the arrangement of starch crystallization regions [13]. Additionally, HMT treatment can make the granules stiffer, heat-resistant, and confer greater hydrophobicity to them [48]. As observed in Figure 6, the swelling power of H-SHC increases with a higher hydrophilic colloid content. For natural starch, starch granules aggregate when hydrophilic colloids cover the granule surface, interacting with the small amount of straight-chain starch on the granule surface. Notably, xanthan gum has a more significant inhibitory effect on the swelling power of starch granules compared to locust bean gum, possibly due to the higher hydrophilicity of locust bean gum, making it more soluble in water and thus leading to the rapid swelling of starch. However, at sufficiently high temperatures, xanthan gum also dissolves in water. Additionally, in the HMT process, straight-chain starch and branched-chain starch form new crystals in the amorphous region of the granule, further inhibiting starch swelling at high temperatures.

As depicted in Figure 6, the solubility of all samples increased significantly with higher solubility temperatures. Natural starch exhibited the highest solubility, which significantly decreased after HMT treatment, likely due to the internal structural rearrangement of starch. The interaction between straight-chain and branched-chain starch molecules led to increased starch densification, rendering the molecules in the starch granules less soluble in water. This reduced the starch’s ability to bind to water and hence its solubility [49]. However, the addition of hydrophilic colloids increased the solubility of the samples, with H-SXG showing particularly significant improvements. The solubility of H-SHC also increased in a concentration-dependent manner with higher hydrophilic colloid content, primarily because increased colloid content inherently possesses better solubility, resulting in increased solubility as the colloid content rises [50].

### 3.8. Analysis of Rheological Properties

#### 3.8.1. Dynamic Rheological Properties

**Figure 7:**
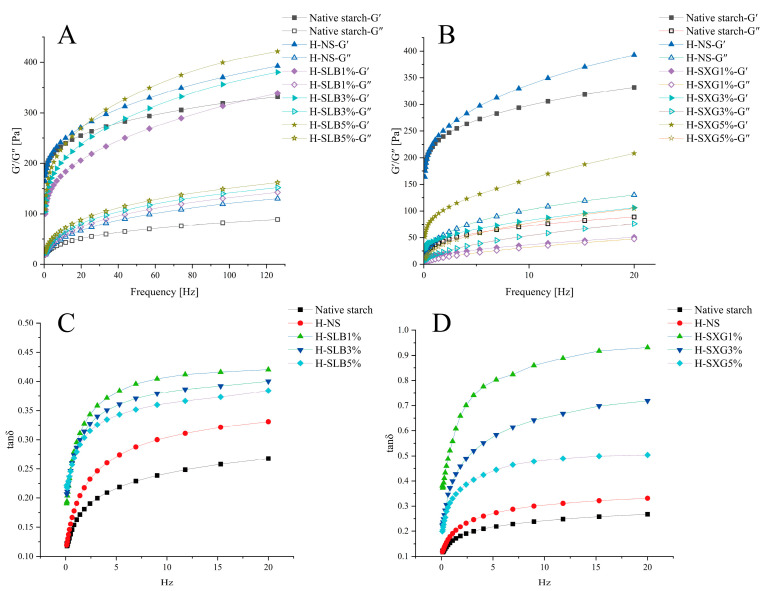
Dynamic rheological properties of natural starch and HNT samples. (**A**) G′, G″ of H-SLB; (**B**) G′, G″ of H-SXG; (**C**) tan δ of H-SLB; (**D**) tan δ of H-SXG.

Figure 7A,B presents the dynamic rheology of natural starch and H-SHC samples. For natural starch, both G′ and G″ increased as the angular frequency increased, indicating that the natural starch gel exhibited weak gel properties [51]. HMT significantly increased both G′ and G″, indicating enhanced gel strength after HMT, which is related to structural changes in starch. All sample gels exhibited frequency dependence, suggesting the presence of a network structure in starch gels [52]. Simultaneously, HMT may lead to starch molecular chain degradation, facilitating the rearrangement of starch molecules into a continuous gel network structure and thereby strengthening the gel [53].

In the frequency range of 0.1 to 20 Hz, different colloids affected G′ and G″ differently. The addition of xanthan gum resulted in the decreased G′ and G″ of the copolymers, while locust bean gum had the opposite effect. The differences in G′ and G″ between the various colloid additions were significant, and both exhibited concentration dependence. When hydrophilic colloids are mixed with starch gels, phase separation between straight-chain starch and hydrophilic colloids occurs in the continuous phase due to thermodynamic incompatibility [52]. Xanthan gum, known for its high viscosity and water-holding capacity, did not increase the stiffness of the mixed gels due to its inability to form an indivisible network structure [54]. The addition of xanthan gum to wheat starch reduced gel properties due to incompatibility between xanthan gum and straight-chain starch in the continuous phase, weakening the gel network. In contrast, locust bean gum increased the G′ and G″ values of starch gels in a concentration-dependent manner. This was because of the incompatibility between locust bean gum and natural starch in the continuous phase, leading to phase separation that increased effective starch concentration by immobilizing water molecules, resulting in a firmer gel [54].

Tan δ, the ratio of G″ to G′, indicates system viscosity, with larger values indicating higher viscosity and vice versa for elasticity. In Figure 7C,D, the tan δ of all samples in the frequency range of 0.1–20 Hz was less than 1, indicating that the samples possessed a weak gel-like structure, with elasticity predominantly dominating over viscosity. Additionally, tan δ decreased with increasing hydrophilic colloid content and displayed concentration dependence, signifying that colloid addition made H-SHC more elastic.

#### 3.8.2. Static Rheology

**Figure 8:**
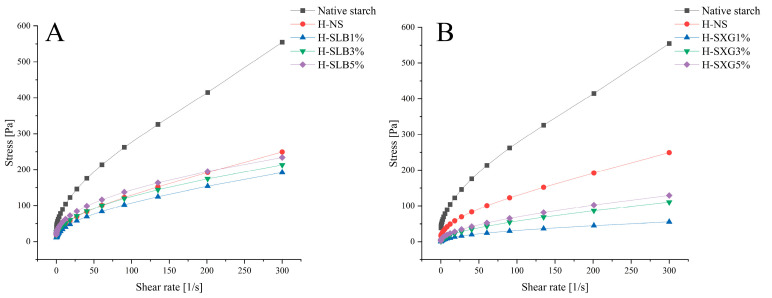
Static rheological properties of natural starch and HMT samples: (**A**) stress of H-SLB; (**B**) stress of H-SXG.

Figure 8 and Table 4 present the steady-state shear rheological properties of the various samples. The shear stress of the samples subjected to HMT was significantly lower than that of natural starch in the shear rate range of 0 to 300 s^−1^. The addition of hydrophilic colloids further reduced the shear stress in the composite system, with this effect being more pronounced in H-SXG. However, as the content of hydrophilic colloid increased, the shear stress also increased. This phenomenon can be explained by the fact that a low concentration of hydrophilic colloids affects the interaction between starch molecules. As the concentration of hydrophilic colloids increases, they form entanglements with starch molecules through hydrogen bonding, increasing the external force required to disrupt the starch paste system.

The Herschel–Bulkley model, widely used to describe the effect of shear rate on fluid rheological properties, yielded fitting coefficients above 0.99 for all samples (Table 5), indicating a strong correlation with the curve fitting. The yield stress (τ_0_) represents the minimum stress required for fluid flow initiation. A larger τ_0_ signifies a greater stress requirement for starch gelatin flow initiation, indicating increased flow resistance. The τ_0_ values of all samples after HMT were lower than those of natural starch, indicating a reduced stress requirement for starch gel flow. Moreover, the τ_0_ values of all samples after HMT were lower compared to natural starch, suggesting that less stress is needed for starch gel flow. This reduction was further pronounced with the addition of hydrophilic colloids, with xanthan gum showing a more substantial decrease than locust bean gum. However, the τ_0_ value increased with increasing hydrophilic colloid content. The *n* values for all samples were less than 1 and decreased with increasing hydrocolloid content [55]. The K-value of H-SLB was significantly higher than that of H-SXG and increased with the addition of hydrophilic colloids. This may be attributed to the inhibitory effect of low concentrations of hydrophilic colloids on natural starch swelling, resulting in reduced thickening of the system. With higher colloid content, natural starch may attach to the hydrophilic segments due to the action of hydrophilic colloids, leading to increased blend consistency [56].

## 4. Conclusions

In vitro digestibility studies indicated that HMT increased the RS content and reduced the GI of starch. The addition of hydrophilic colloids further improved the antidigestibility of natural starch, with locust bean gum exhibiting better antidigestibility than xanthan gum. This improvement was associated with a denser crystal structure. XRD and FT-IR analyses revealed hydrogen bonding interactions between colloids and starch. The addition of hydrophilic colloids increased the crystallinity and short-range ordering of the samples compared to HMT alone, suggesting that hydrophilic colloids contributed to a denser crystal structure of starch granules. Xanthan gum demonstrated a similar effect to locust bean gum in a concentration-dependent manner. HMT led to the decreased solubility and swelling of starch, but the addition of hydrophilic colloids slightly increased solubility and swelling, with xanthan gum having a more pronounced effect in a concentration-dependent manner. Results from RVA and DSC indicated that HMT reduced the final viscosity and disintegration value of starch while increasing the initial pasting temperature of the starch.

## Figures and Tables

**Table 1:** In vitro digestion parameters of natural starch and HMT samples.

	RDS (%)	SDS (%)	RS (%)	C_∞_ (%)	HI	eGI	R^2^
Native starch	33.18 ± 0.52 c	58.4 ± 0.9 d	8.42 ± 0.39 a	95.83 ± 0.99 g	94.89 ± 0.6 g	89.99 ± 0.65 g	0.9987
H-NS	41.71 ± 0.6 d	43.87 ± 1.22 c	14.42 ± 0.73 b	86.96 ± 0.13 f	88.94 ± 0.3 f	84.86 ± 0.29 f	0.9963
H-SLB1%	28.42 ± 1.27 b	40.96 ± 1.54 abc	30.62 ± 2.59 cd	72.53 ± 0.35 e	72.45 ± 0.31 d	70.65 ± 0.19 d	0.9948
H-SLB3%	26.47 ± 2.96 ab	36.77 ± 5.08 a	36.76 ± 1.44 e	66.26 ± 0.32 b	67.85 ± 0.27 b	66.68 ± 0.94 b	0.9939
H-SLB5%	24.66 ± 1.92 a	36.98 ± 2.43 ab	38.36 ± 3.69 e	64.56 ± 0.1 a	65.45 ± 0.5 a	64.62 ± 0.59 a	0.9928
H-SXG1%	32.19 ± 0.95 c	42.6 ± 2.51 bc	25.21 ± 1.62 c	77.36 ± 0.31	78.13 ± 0.06 e	75.54 ± 0.11 e	0.9971
H-SXG3%	25.94 ± 2.18 ab	42.92 ± 3.58 c	31.14 ± 1.67 d	71.5 ± 0.15 d	72.3 ± 0.58 d	70.52 ± 0.12 d	0.9932
H-SXG5%	27.19 ± 1.16 ab	38.43 ± 4.2 abc	34.39 ± 4.11 de	68.66 ± 0.49 c	69.61 ± 0.14 c	68.2 ± 0.43 c	0.9944

Mean ± S.D. followed by different letters within the same column are significantly different (*p* ≤ 0.05).

**Table 2:** Natural starch with HMT samples R_1047/1022_ and RC.

	RC (%)	R_1047/1022_
Native starch	33.72 ± 0.31 d	0.8657 ± 0.0026 a
H-NS	26.88 ± 0.54 a	0.9377 ± 0.005 b
H-SLB1%	27.12 ± 0.42 a	1.0182 ± 0.0068 c
H-SLB3%	32.64 ± 0.56 c	1.0194 ± 0.0179 c
H-SLB5%	33.17 ± 0.48 cd	1.0564 ± 0.0127 c
H-SXG1%	28.62 ± 0.37 b	1.0264 ± 0.0769 c
H-SXG3%	33.29 ± 0.33 cd	1.023 ± 0.0954 c
H-SXG5%	33.24 ± 0.52 cd	1.025 ± 0.0743 c

Mean ± S.D. followed by different letters within the same column are significantly different (*p* ≤ 0.05).

**Table 3:** Pasteurization characteristics of natural starch and HMT samples.

Test	Peak Viscosity (cP)	Trough Viscosity (cP)	Breakdown (cP)	Final Viscosity (cP)	Setback (cP)	Pasting Temperature (°C)
Native starch	2536 ± 8.91 g	1585 ± 14.21 g	951 ± 11.06 e	2851 ± 5.58 h	1266 ± 12.66 h	73.47 ± 0.47 a
H-NS	1062 ± 13.99 f	947 ± 11.16 f	115 ± 11.36 d	1298 ± 8.93 g	351 ± 3.51 f	75.61 ± 0.31 b
H-SLB1%	774 ± 12.63 c	682 ± 11.49 c	92 ± 5.25 c	936 ± 8.25 d	254 ± 2.54 d	78.32 ± 0.48 d
H-SLB3%	874 ± 14.17 d	773 ± 7.56 d	101 ± 9.37 cd	1099 ± 14.63 e	326 ± 3.26 e	79.12 ± 0.45 e
H-SLB5%	974 ± 7.6 e	864 ± 10.41 e	110 ± 8.64 d	1262 ± 12.4 f	398 ± 3.98 g	79.92 ± 0.3 f
H-SXG1%	365 ± 6.1 a	358 ± 13.89 a	7 ± 2.78 a	428 ± 9.12 a	70 ± 0.7 a	77.34 ± 0.47 c
H-SXG3%	427 ± 6.87 b	403 ± 9.27 b	24 ± 6.96 b	525 ± 14.46 b	122 ± 1.22 b	78.64 ± 0.42 de
H-SXG5%	433 ± 13.85 b	407 ± 14.66 b	26 ± 8.02 b	604 ± 12.91 c	197 ± 1.97 c	78.83 ± 0.47 de

Mean ± S.D. followed by different letters within the same column are significantly different (*p* ≤ 0.05).

**Table 4:** Thermal properties of natural starch and HMT samples.

	T_0_/°C	T_p_/°C	T_c_/°C	T_c_ − T_0_/°C	ΔH/(J/g)
Native starch	63.34 ± 0.17 a	71.68 ± 0.15 a	80.61 ± 0.38 a	17.27 ± 0.5 a	15.98 ± 0.08 e
H-NS	66.56 ± 0.24 bc	75.11 ± 0.06 b	85.92 ± 0.42 b	19.36 ± 0.4 b	13.38 ± 0.5 a
H-SLB1%	66.9 ± 0.11 cd	75.82 ± 0.29 c	86.91 ± 0.43 c	20.01 ± 0.09 c	13.72 ± 0.12 ab
H-SLB3%	66.67 ± 0.44 bcd	77.175 ± 0.13 d	89.46 ± 0.36 d	22.79 ± 0.09 d	14.64 ± 0.16 c
H-SLB5%	66.42 ± 0.2 b	76.96 ± 0.29 d	89.4 ± 0.17 d	22.98 ± 0.3 de	15.26 ± 0.4 d
H-SXG1%	67.09 ± 0.2 d	75.89 ± 0.33 c	87.19 ± 0.46 c	20.1 ± 0.43 c	13.21 ± 0.47 a
H-SXG3%	67.68 ± 0.28 e	78.475 ± 0.1 e	91.15 ± 0.19 e	23.47 ± 0.3 e	14.07 ± 0.33 b
H-SXG5%	68.2 ± 0.22 f	79.005 ± 0.25 f	91.72 ± 0.29 e	23.52 ± 0.41 e	15.23 ± 0.18 d

Mean ± S.D. followed by different letters within the same column are significantly different (*p* ≤ 0.05).

**Table 5:** Herschel–Bulkley model parameters of natural starch and HMT samples.

	τ_0_	K	*n*	R^2^
Native starch	40.679 ± 0.2 a	11.167 ± 0.06 b	0.667 ± 0.04 a	0.9987
H-NS	15.789 ± 0.27 b	6.702 ± 0.16 e	0.619 ± 0.03 ab	0.9993
H-SLB1%	8.293 ± 0.26 e	7.972 ± 0.15 d	0.549 ± 0.03 cd	0.9998
H-SLB3%	14.953 ± 0.24 d	9.573 ± 0.1 c	0.531 ± 0.02 cde	0.9989
H-SLB5%	16.766 ± 0.09 c	13.548 ± 0.1 a	0.486 ± 0.03 de	0.9999
H-SXG1%	0.03 ± 0.01 h	3.048 ± 0.08 h	0.507 ± 0.02 e	0.9999
H-SXG3%	0.218 ± 0.09 g	4.104 ± 0.18 f	0.575 ± 0.04 bc	0.9997
H-SXG5%	3.147 ± 0.14 f	4.717 ± 0.14 g	0.575 ± 0.03 bc	0.9994

Mean ± S.D. followed by different letters within the same column are significantly different (*p* ≤ 0.05).

## Data Availability

The data used to support the findings of this study can be made available by the corresponding author upon request.
